# Increasing saving intentions through leaderboards: A gamification approach

**DOI:** 10.1371/journal.pone.0249283

**Published:** 2021-04-14

**Authors:** Yi Zhang, Femke van Horen, Marcel Zeelenberg

**Affiliations:** 1 Department of Experimental and Applied Psychology, Vrije Universiteit Amsterdam, Amsterdam, The Netherlands; 2 Marketing Department, Vrije Universiteit Amsterdam, Amsterdam, The Netherlands; 3 Department of Social Psychology, Tilburg University, Tilburg, The Netherlands; Sheffield Hallam University, UNITED KINGDOM

## Abstract

Saving money is important but challenging. To spur financial saving intentions, we propose a new strategy—gamification. Specifically, we investigate the effectiveness of competitive leaderboards in increasing individuals’ saving intentions. The results of two studies (total *N* = 618) show consistently that people’s saving intentions are higher when presented with a leaderboard than when not. Further, as leaderboards elicit social comparison, we explore whether the height of the comparison standard and individuals’ social comparison orientation moderate the effect. We find that the effect of leaderboards on saving intentions is more pronounced when people compare with a higher (as compared to a lower) standard (Study 1), but that the effect is not influenced by individuals’ social comparison orientation (Study 2). Taken together, this research provides a new and simple-to-implement strategy to facilitate saving intentions in order to help improve people’s financial well-being.

## Introduction

People save too little. In the United States, one-quarter of the non-retired workers and 13% of the people who are about to retire (age 60 and older) have no retirement savings or pension [1]. Likewise, about 27% of European respondents of the ING international savings survey have no savings at present and 42% of Europeans with savings have no more than three month’s take-home pay put aside [2]. Meanwhile, household saving rates have dropped worldwide. In recent years, household savings in the United States have fallen from 13.11% of household disposable income in 1970 to 6.90% in 2017, as OECE shows [3]. In the European Union, household savings have declined from 6.82% of household disposable income in 1999 to 3.25% in 2017 [3]. This downward trend is expected to continue in the future [4]. Lack of savings is associated with adverse consequences, such as severe financial hardship, poor health, and generally lower life quality [5]. Even though people are aware of the importance of saving, they have a hard time doing so.

Explanations for why people fail to save their money often focus on people’s social circumstances (e.g., education, familial upbringing, poverty) or their personality characteristics (e.g., low self-control, impulsivity). These factors are relatively difficult to change [6, 7]. More recently, researchers have proposed small psychological shifts in people’s mindset (e.g., feelings of power; [8]) or changes in their choice environment (e.g., the Save More Tomorrow program; [9]) to facilitate saving intentions and behavior. In the current research, instead of focusing on eliminating the temptation to spend, we investigate a new and easy-to-implement strategy that could motivate people to save by making it fun: gamification. We examine whether gamification (applying game-like elements or principles to a non-game context to stimulate desirable behaviors; [10–12]), specifically competitive leaderboards, can increase individuals’ intentions to save. Also, we explore under which conditions this form of gamification has the highest impact on saving intentions. As leaderboards have a strong competitive element in which people are confronted with their position as compared to that of others, we examine whether the comparison standard and individuals’ social comparison orientation [13] impact the effectiveness of leaderboards on the intentions to save.

### Why saving is difficult

Saving is defined as “refraining from spending the whole current income on consumption” ([14], p. 172). According to Katona ([15], p. 4–5), “the principal reason for saving is to be prepared for rainy days. Because the future is uncertain, reserve funds prepare for possible adversity.” People can save to achieve short-term or long-term goals, such as purchasing a washing machine or having sufficient money for retirement. Despite people’s willingness to save and their good intentions, however, the majority of people fail to save. On an impulsive moment, instead of depositing money in one’s savings account, one may buy an expensive pair of shoes or treat one’s partner for a romantic dinner at a fancy restaurant.

Previous studies have proposed several factors that might explain why people cannot always stick to their saving goals. Demographically, people’s age, the size of their household, educational level, and the environment they grow up in, have often been related to people’s saving behavior [16, 17]. For instance, people who grow up in low (versus high) socioeconomic status environments, where resources are scarce, are less likely to save for future purposes, are more impulsive, and show a higher preference for short-term and high-interest loans [17, 18]. Psychologically, the difficulty of resisting temptations is one of the most important explanations for why humans have problems saving [19, 20]. Even if people are capable of calculating the optimal amount to save this year for a good retirement later, willpower is needed to resist the temptation of current consumption. Such self-control problems are often explained through present bias [21]. Present bias refers to generally over-valuing immediate rewards at the expense of long-term benefits. For example, individuals prefer to receive 10 euros today over receiving 15 euros in two weeks. Considering that saving requires sacrificing immediate rewards in return for future benefits, saving can be difficult for people prone to present bias. Indeed, evidence shows that present bias is related to fewer savings, lower bank card balance, and more credit card debt [22, 23].

Given that saving is challenging for many, what can be done to help people improve their saving behavior? Prior literature has suggested several strategies, including goal setting [24, 25], earmarking (labeling money for a particular purpose to be deposited on a separate account; [26]), and avoidance of tempting stimuli [27]. In addition, several programs have been set up to increase people’s savings, for instance through putting a proportion of salary automatically into retirement funds (e.g., [9, 28]) or programs in which a transaction is automatically rounded up by banks and the difference is put into a saving account [29].

Commitment devices have also been put forward as a useful tool to prompt savings. Commitment devices are mechanisms that impose restrictions on one’s choices in order for people to refrain from impulsive behaviors and to limit choices to those that reflect people’s long-term goals [20]. An example of a conventional commitment device to promote savings is a piggy bank, which, when locked, spurs people to cut down unnecessary consumption and save money [30]. Importantly, commitment devices must be voluntary to be used and tie consequences to people’s future selves’ failures. There are several successful examples of commitment devices that encourage individuals’ savings: in the SEED (Save, Earn, Enjoy Deposits) program, the account balance only became available after specific dates or when people reached specific pre-set goals [31].

Despite their valuable insights, the strategies above are most often based on restrictions to resist temptations (taking away the temptation to spend). In the current research, we explore a new strategy to spur saving intentions by turning saving into a fun and competitive experience. Specifically, we aim to test whether gamification can encourage people to save more.

### Gamification and save intentions

Gamification can be defined by the use of game elements in a non-game context to promote desired behaviors [10, 12]. Game elements usually include (digital) points and badges, leaderboards, rankings, and progress tracking. For example, fitness trainers may give badges to those who achieve daily goals to motivate them to continue exercising. The mechanism in which these game elements function is to induce competition, to provide feedback, and to grant rewards [11]. Gamification has been an increasingly popular tool to induce behavioral change across various domains, such as education, health, crowdsourcing, and sustainability (for a review see [11, 32]). For example, gamification has been shown to increase grades and improve knowledge [33], to enhance physical exercise and boost healthy eating [34, 35], and to facilitate energy-saving and waste recycling behaviors [36, 37]. Because gamification is mostly fun and engaging, it increases enjoyment and motivates individuals to attain goals [38–41].

People experience difficulties saving because they are present-biased and are easily tempted. Instead of focusing on how to resist temptations—the main focus of previous research—we investigate in the current research, whether saving intentions can be improved through gamification. In doing so, we highlight one element of gamification: leaderboards. By providing a ranking of people based on their performance, a fundamental part of human nature is stimulated—the motivation to compete [42]. Competition has been found to be very useful in creating deeper involvement and motivation among people [43]. Besides, the competitive process itself can spur excitement, which makes the competition fun and attractive, and therefore, is appealing to a majority of people [44, 45]. Hence, we hypothesize that competitive leaderboards increase individuals’ financial saving intentions. Because leaderboards confront people with their positions as compared to those of others, we explore how social comparison moderates the effect.

### The role of social comparison in leaderboards

By applying leaderboards and rankings, gamification stimulates competition with others through the process of social comparison. After being confronted with one’s position on a leaderboard, people naturally compare themselves with others and become aware of their relative performance. According to Festinger’s social comparison theory [46], people often compare themselves with others to gain accurate self-knowledge. By comparing themselves with inferior others (downward comparison), people can additionally maintain or boost positive self-views [47]. By comparing with superior others (upward comparison), people can obtain information about their relative standing in a particular domain and whether to improve oneself. Such an upward comparison has been found to motivate people to do better, but it may additionally threaten individuals’ self-views [48].

Two factors might influence the effectiveness of upward comparisons on performance improvement: the comparison standard (with whom individuals compare themselves) and individuals’ social comparison orientation [13]. The standard of upward comparison can be high (as compared to low), indicating a larger gap between the standard and the individual. High standards might increase the motivation to perform better. For example, many leaders set higher performance standards to raise employees’ performance [49]. However, upward comparisons only motivate people to improve themselves when they perceive the upward standard to be attainable [48]. Then, too high standards of upward comparison might decrease the motivation to perform better.

Aside from the standard of comparison, individuals’ social comparison orientation (SCO) might also affect the effectiveness of upward comparisons [13]. Social comparison orientation refers to a chronic individual difference in the tendency to compare oneself with others. Individuals high in SCO compare themselves more frequently with others and are affected more by these social comparisons, more so than individuals low in SCO [13, 50]. In this case, the positive effect of upward comparisons on the motivation to improve oneself might be more pronounced among people high in SCO relative to people low in SCO. However, compared with people low in SCO, people with high SCO also display more negative affectivity, are more uncertain about themselves [51, 52], and feel more regret when decisions go awry [53]. In this case, upward comparisons might damage high-SCO people’s self-views, lower their confidence in reaching the superior standard, and decrease the motivation to attain the upward standard. That is to say, it is also possible that the positive effect of upward comparison on motivation to perform better might be weakened among people with high SCO relative to people with low SCO.

Thus, the standard of comparison and individuals’ SCO are both expected to influence the effectiveness of upward comparisons on performance improvement, which is the core of competitive leaderboards. In the current research, therefore, we explore the extent to which the predicted positive effect of leaderboards on individuals’ financial saving intentions is moderated by the standard of upward comparison (high versus low) and people’s SCO.

### The present research

This research is, to the best of our knowledge, the first to investigate the effectiveness of competitive leaderboards on individuals’ financial saving intentions. Study 1 investigates whether leaderboards increase people’s intentions to save. This study explores further whether the standard of upward comparison (low versus high) moderates this effect. Study 2 aims to replicate the effect of leaderboards on saving intentions in Study 1. In addition, this study explores whether individuals’ SCO moderates this effect. In both studies, we used the presence of a leaderboard versus no leaderboard as the leaderboard manipulation and measured individuals’ saving intentions for retirement.

Gamification has been identified to have generally favorable effects on behavioral change across different domains, but not yet for saving behavior. In the present research, we focus on competitive leaderboards, a specific form of gamification, as they can be easily implemented on online platforms. We believe that leaderboards potentially provide a convenient, attractive, and effective way to increase individuals’ saving intentions. We decide to focus here only on leaderboards, as it is unlikely that the different forms of gamification work in the same way. Hence, these need to be studied separately.

## Study 1: Leaderboards and comparison standards

Study 1 tested whether merely displaying a competitive leaderboard increases people’s intentions to save. In addition, we explored whether this effect is moderated by the standard of upward comparison (whether one is low versus high on the leaderboard). More specifically, participants read that they would receive a surplus of $100 on their monthly salary and were asked how much they would want to save for their retirement. We decided to focus on retirements, as saving for this long-term goal has been proven to be particularly difficult, but generally found to be important [54]. To better understand the impact of competitive leaderboards on saving intentions, we measured the intended saving amount before and after the manipulation (leaderboard condition vs. no-leaderboard condition) and calculated two indicators of saving intentions: absolute savings and relative savings. Absolute savings is the difference between the intended savings before and after the manipulation, reflecting the absolute growth of intended savings. Relative savings is the ratio of two intended savings, reflecting the relative growth of intended savings.

### Method

This research complied with the research ethical guidelines of the School of Business and Economics at Vrije Universiteit Amsterdam and was declared an approval (reference number: SBE8/4/2020fhn740).

#### Participants and design

Two hundred and eighty-one US-based Amazon Mechanical Turk workers were recruited in return for a small monetary compensation ($0.5 for 5 minutes on average; required N = 256, based on a power analysis with η_p_^2^ = .03, α = .05, power = .80; [55]). They were randomly allocated to a 2 (Leaderboards: no, yes) × 2 (Standards of upward comparison: low, high) factorial design. Participants who failed the attention check (n = 19), did not complete the questionnaire (n = 12), entered a value of zero or nothing as Saving 1 (n = 7; in order to calculate the percentage increase for the low and high standards, the value entered for Saving 1 had to be above zero), and those who revealed to be outliers in their saving amount (+4 SD (in this sample amounts higher than $185.35 (Saving 1) and $255.00 (Saving 2)), n = 2) were excluded, leaving 241 participants (114 women, M_age_ = 36.12, SD = 11.51) for the analyses.

#### Procedure and measures

Participants completed this study online via Mturk. In a brief introduction, participants read they would take part in a study investigating saving behavior. Participants indicated whether they agreed on the informed consent by clicking a button (Yes/No) in the beginning of the online questionnaire. After agreeing on the informed consent, all participants were asked to imagine that they wanted to start saving money for their retirement. They read that from now on they would receive each month $100 on top of their regular salary, which they could use to save up for retirement. Participants then indicated (in rounded dollars) how much of the $100 extra salary they were willing to save for retirement, which was referred to as Saving 1. Participants then read that their colleagues also received $100 extra salary each month, which they could save for their retirement too. Next, participants were randomly assigned to either the leaderboard condition or the no-leaderboard condition.

In the no-leaderboard condition, participants read:

“During a lunch-break, you talk with some colleagues about the importance of saving for your retirement. Your colleagues indicate that they also want to save each month for their retirement. After several weeks, you and your colleagues have lunch and talk about how much each of you has saved this month. Bob saved most, $X this month.”

In the leaderboard condition, they read thereafter:

“Together you and your colleagues decide that you will challenge each other to save more by turning it into a game. Every month a ranking board is presented on the wall to see how everyone is doing. This month you are ranked number five on the list. The best saver was Bob, and he has saved $X this month (see S1 File for an image of the ranking board and more scenario details).”

In both the no-leaderboard and leaderboard scenarios, Bob’s saving was either 1.10 times participants’ Saving 1 (low standard of upward comparison) or 1.60 times participants’ Saving 1 (high standard of upward comparison). To distinct a low standard and a high standard, the two standards should be distanced from each other [48]. Thus, we chose 1.10 and 1.60 separately. Meanwhile, 1.60 seemed not unattainable and thus could make the scenario more realistic. After reading the scenario, participants indicated (in rounded dollars) how much they would save next month (Saving 2). Participants also reported their willingness to save for retirement on a scale ranging from 1 (very little) to 7 (very much). Next, participants answered two manipulation check questions (“Did the scenario described, in which you and your colleagues saved for retirement, involve a game?”; 1 = *Strongly disagree*, 7 = *Strongly agree*; “Compared to my saving amount, the investment of Bob was”; 1 = *Much lower*, 7 = *Much higher*) and one attention check question (“For data-quality reasons, now please check the first box from the left”). Participants were then asked to think back at the saving scenario and to report their motivation to save on three questions (“I feel motivated to save more after reading the scenario”; “I feel competent to save more the next month”; “I feel a sense of accomplishment by saving”; all on 1 = *Strongly disagree*, to 7 = *Strongly agree*). Since the willingness to save was highly correlated with the three items of motivation to save, these four items were averaged into one motivation to save variable (α = .79). We measured motivation to save as a potential mediator of the effect of leaderboard on saving intentions, but this did not satisfy all the requirements of meaningful mediation according to Pieters [56]. Therefore, we would not further report it.

Then, as control variables, participants responded to questions measuring their attitude towards saving in general (1 = *Very negative*; 7 = *Very positive*), their satisfaction with their current savings (1 = *Very dissatisfied*; 7 = *Very satisfied*), and how regularly they save (“Do you save on a regular basis (e.g., weekly, monthly)”; 1 = *Yes*; 2 = *No*). Finally, participants reported their demographics (gender, age, employment status, annual income, and household composition) and were thanked for their participation.

### Results

#### Manipulation check

The leaderboard and comparison standard manipulations were successful. Participants in the leaderboard condition agreed to a higher extent that the scenario they read involved a game (M = 5.98, SD = 1.60) than participants in the no-leaderboard condition (M = 1.98, SD = 1.46), t(239) = –20.14, p < .001, d = 2.61. Also, in the high-comparison-standard condition, participants indicated that Bob’s saving amount was higher (M = 5.16, SD = 1.31) than in the low-comparison-standard condition (M = 4.42, SD = 1.01), t(215.99) = –4.83, p < .001, d = 0.63.

#### Intentions to save

**Fig 1:**
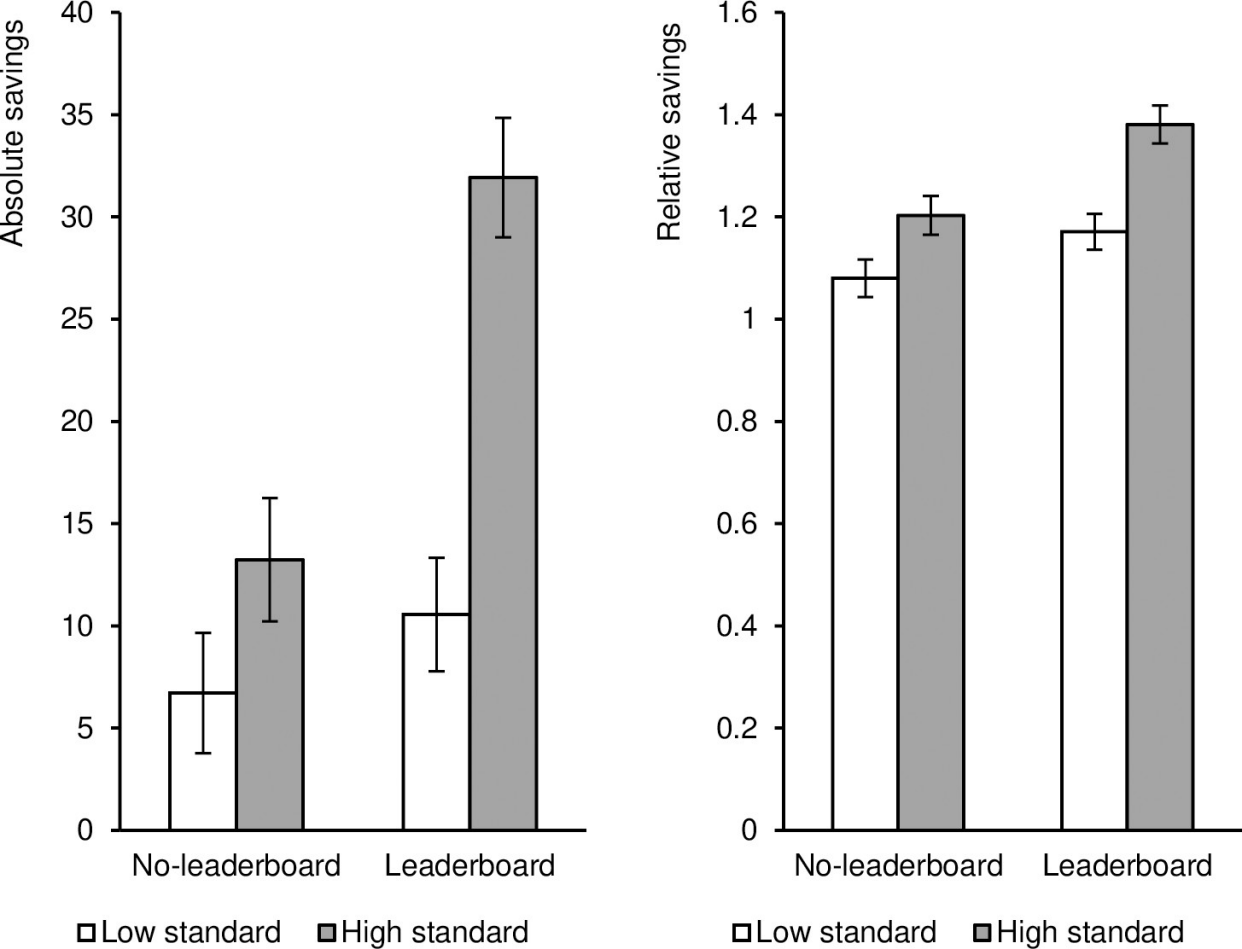
**Absolute savings (left panel) and relative savings (right panel) as a function of the leaderboard and the standard of upward comparison (low vs. high).** (Error bars indicate +/–1 Standard Error of the Mean).

On absolute savings, a 2 (leaderboard) × 2 (comparison standard) ANOVA revealed, as predicted, a significant main effect of the leaderboard, F(1, 237) = 14.96, p < .001, η_p_^2^ = .06. Absolute savings were higher in the leaderboard condition (M = 20.74, SD = 26.28) than in the no-leaderboard condition (M = 9.89, SD = 20.99). There was also a significant main effect of the comparison standard, F(1, 237) = 22.88, p < .001, η_p_^2^ = .09. Absolute savings were higher in the high-comparison-standard condition (M = 22.91, SD = 29.71) than in the low-comparison-standard condition (M = 8.74, SD = 15.56). In addition, there was a significant interaction effect between the leaderboard and the comparison standard, F(1, 237) = 6.49, p = .01, η_p_^2^ = .03 (see Fig 1, left panel).

Follow-up simple effect tests indicated that, in the low-comparison-standard condition, absolute savings did not differ between the no-leaderboard (*M* = 6.71, *SD* = 16.80) and leaderboard conditions (*M* = 10.56, *SD* = 14.25), *F*(1, 237) = 0.90, *p* = .34, η_p_^2^ = .004. However, in the high-comparison-standard condition, absolute savings were higher in the leaderboard condition (*M* = 31.93, *SD* = 31.56) than in the no-leaderboard condition (*M* = 13.23, *SD* = 24.36), *F*(1, 237) = 19.85, *p* < .001, η_p_^2^ = .08. In the no-leaderboard condition, the low- and high-comparison-standard conditions did not differ, *F*(1, 237) = 2.39, *p* = .12, η_p_^2^ = .01. However, in the leaderboard condition, absolute savings in the high-comparison-standard condition was higher than in the low-comparison-standard condition, *F*(1, 237) = 28.13, *p* < .001, η_p_^2^ = .11.

On relative savings, consistent with the results for absolute savings, the ANOVA on relative savings revealed a significant main effect of the leaderboard, *F*(1, 237) = 13.48, *p* < .001, η_p_^2^ = .05; relative savings were higher in the leaderboard condition (*M* = 1.27, *SD* = 0.31) than in the no-leaderboard condition (*M* = 1.14, *SD* = 0.28). Also, the main effect of the comparison standard was significant, *F*(1, 237) = 20.83, *p* < .001, η_p_^2^ = .08; relative savings were higher in the high-comparison-standard condition (*M* = 1.30, *SD* = 0.36) than in the low-comparison-standard condition (*M* = 1.13, *SD* = 0.21). The interaction between the leaderboard and the comparison standard was not significant, *F*(1, 237) = 1.41, *p* = .24, η_p_^2^ = .01 (Fig 1, right panel).

As the pattern of results is similar to the absolute savings, further simple effect analyses were conducted to get deeper insight into the effect of leaderboards separately for the two comparison standard conditions, despite the nonsignificant interaction. The results showed that, in the low-comparison-standard condition, relative savings did not differ between the no-leaderboard (*M* = 1.08, *SD* = 0.19) and leaderboard conditions (*M* = 1.17, *SD* = 0.21), *F*(1, 237) = 3.21, *p* = .08, η_p_^2^ = .01. In the high-comparison-standard condition, relative savings were significantly higher in the leaderboard condition (*M* = 1.38, *SD* = 0.36) than in the no-leaderboard condition (*M* = 1.20, *SD* = 0.34), *F*(1, 237) = 11.38, *p* < .001, η_p_^2^ = .05. Besides, in both no-leaderboard and leaderboard conditions, relative savings of high-comparison-standard condition were always significantly higher than that of low-comparison-standard condition (for the no-leaderboard condition: *F*(1, 237) = 5.46, *p* = .02, η_p_^2^ = .02; for the leaderboard condition: *F*(1, 237) = 17.30, *p* < .001, η_p_^2^ = .07).

Additionally, we repeated the above analyses, this time including the outliers on the amount saved (Savings 1 and 2). The results remained the same as the above. On absolute savings and relative savings, there was a main effect of leaderboard, *p*s < .04. Furthermore, the interaction between leaderboard and comparison standard was significant for absolute savings (*F*(1, 239) = 4.54, *p* = .03, η_p_^2^ = .02) but not for relative savings (*F*(1, 239) = 2.30, *p* = .13, η_p_^2^ = .01), see S2 File for further details).

Next, we ran ANCOVAs, taking into account several control variables separately. This analysis revealed a significant effect of general attitude toward saving (*F*(1, 236) = 5.36, *p* = .02, η_p_^2^ = .02), of employment status (*F*(1, 235) = 5.47, *p* = .02, η_p_^2^ = .02), and of age (*F*(1, 235) = 3.01, *p* = .08, η_p_^2^ = .01) on absolute savings. Furthermore, an effect of general attitude towards saving (*F*(1, 236) = 6.12, *p* = .01, η_p_^2^ = .03), of employment status (*F*(1, 235) = 4.92, *p* = .03, η_p_^2^ = .02), and of household composition (*F*(1, 235) = 3.77, *p* = .05, η_p_^2^ = .02) on relative savings was found. Importantly, after inclusion of the control variables, the pattern of results remained the same. None of the other control variables (satisfaction with current savings and annual income) did explain any of the variances of both saving measures (*p*s > .12).

Finally, exploratory analyses found that the regularity of saving influenced the effects of leaderboards and comparison standards on saving intentions (on absolute savings: *F*(3, 233) = 4.67, *p* = .003, η_p_^2^ = .06; on relative savings: *F*(3, 233) = 7.74, *p* < .001, η_p_^2^ = .09; a sensitivity power analysis with α = .05, 80% power, *N* = 241, suggested that the smallest detectable effect size with this simple was η_p_^2^ = .03). This three-way interaction revealed that the main effects of leaderboards and comparison standards and the interaction between leaderboards and comparison standards on saving intentions was found for participants who save regularly (on absolute savings: *p*s < .001; on relative savings: *p*s < .001), but not for participants who do not save regularly (on absolute savings: *p*s > .13; on relative savings: *p*s > .08; see S3 File for analysis details). We address this finding further in the General Discussion.

### Discussion

These results reveal that competitive leaderboards can have a positive effect on people’s retirement saving intentions: Merely presenting a leaderboard increased participants’ absolute and relative saving intention. Furthermore, the results also show that the standard of upward comparison moderates this effect: the positive influence of leaderboard on absolute savings was strengthened when the comparison standard was high (60% higher than participants’ firstly intended saving amount) as compared to when it was low (10% higher than participants’ firstly intended saving amount).

## Study 2: Leaderboards and social comparison orientation

Study 2 tested the robustness of the findings of Study 1. Additionally, we explored whether individuals’ social comparison orientation [13] moderates the effect of leaderboards on saving intentions.

### Method

#### Participants and design

Given the observed effect sizes in Study 1, a power analysis (η_p_^2^ = .02, α = .05, power = .80) suggested a sample size of 387 people [55]. Four hundred and seven (+/- 10% extra to allow for drop out) US-based MTurkers participated in return for a small monetary compensation ($0.50 for 5 minutes on average) were randomly assigned to either the no-leaderboard or leaderboard condition. Similar to Study 1, we excluded participants who did not complete the entire questionnaire (n = 7), failed the attention check (n = 1) or failed to fill out the survey on computer or laptop (as specifically instructed in the beginning of the survey, n = 9), entered a value of zero or nothing in Saving 1 (n = 6; as a value of 0 makes the manipulation of comparison standards inoperative), or were indicated as outliers (+ 4 SD (in this sample amounts higher than $186.28 (Saving 1) and $268.15 (Saving 2)), n = 7). This left us with 377 participants for the analyses (180 women, M_age_ = 38.06, SD = 10.73).

#### Materials

As leaderboard manipulation, this study used the same scenarios of the no-leaderboard and leaderboard conditions with the high comparison standard as used in Study 1. In both the no-leaderboard and leaderboard conditions, Bob saved 60% more money than participants’ Saving 1.

The 11-item Iowa-Netherlands Comparison Orientation Scale (INCOM) [13] measured participants’ social comparison orientation (e.g., “I often compare myself with others with respect to what I have accomplished in life”, “I am not the type of person who compares often with others” [reverse coded], and “I never consider my situation in life relative to that of other people” [reverse coded]; 1 = *Strongly disagree* to 5 = *Strongly agree*). A higher average score indicated a higher inclination to compare with others (*M* = 3.35, *SD* = 0.75, α = .89).

#### Procedure and measures

After agreeing on the informed consent by clicking a button, participants first completed the SCO questionnaire followed by an attention check question (“For data-quality reasons, please drag the slider to the number 43”). The further procedure was mostly the same as in the high-comparison-standard condition of Study 1, except that this study used a single item to directly measure participants’ motivation to save for retirement immediately after indicating their Saving 2 (“After reading the scenario above, how motivated would you be to save for retirement?”; 1 = Not motivated at all, 7 = Very motivated). Because this potential mediator did not satisfy all the requirements of meaningful mediation according to Pieters [56], we did not explore it any further. Then, participants were asked the same manipulation check question as in Study 1 and to indicate their perceived importance of saving for retirement (“How important do you think it is to start saving for retirement as early as possible”; 1 = Not important; 7 = Very important) and of saving generally (“In general, how important do you think it is to save”; 1 = Not important; 7 = Very important), their attitudes toward saving (“In general, what is your attitude towards saving”; 1 = Negative; 7 = Positive), and the amount of saving each month (“How much money do you save each month?”, choose of one out of eight options: $0, $0–10, $10–25, $25–50, $50–100, $100–250, $250–500,>$500). Finally, participants reported their demographics, including gender, age, current financial situation (“How would you describe your own financial situation?”, Very poor–Very wealthy), and parental financial situation (“How would you describe the financial situation of your parents?”, Very poor–Very wealthy).

### Results

#### Manipulation check

The leaderboard manipulation was successful. Participants in the leaderboard condition indicated more often that the scenario involved a game (M = 5.99, SD = 1.64), than participants in the no-leaderboard condition (M = 1.39, SD = 1.06) t(317.37) = –32.34, p < .001, d = 3.34.

#### Intentions to save

**Fig 2:**
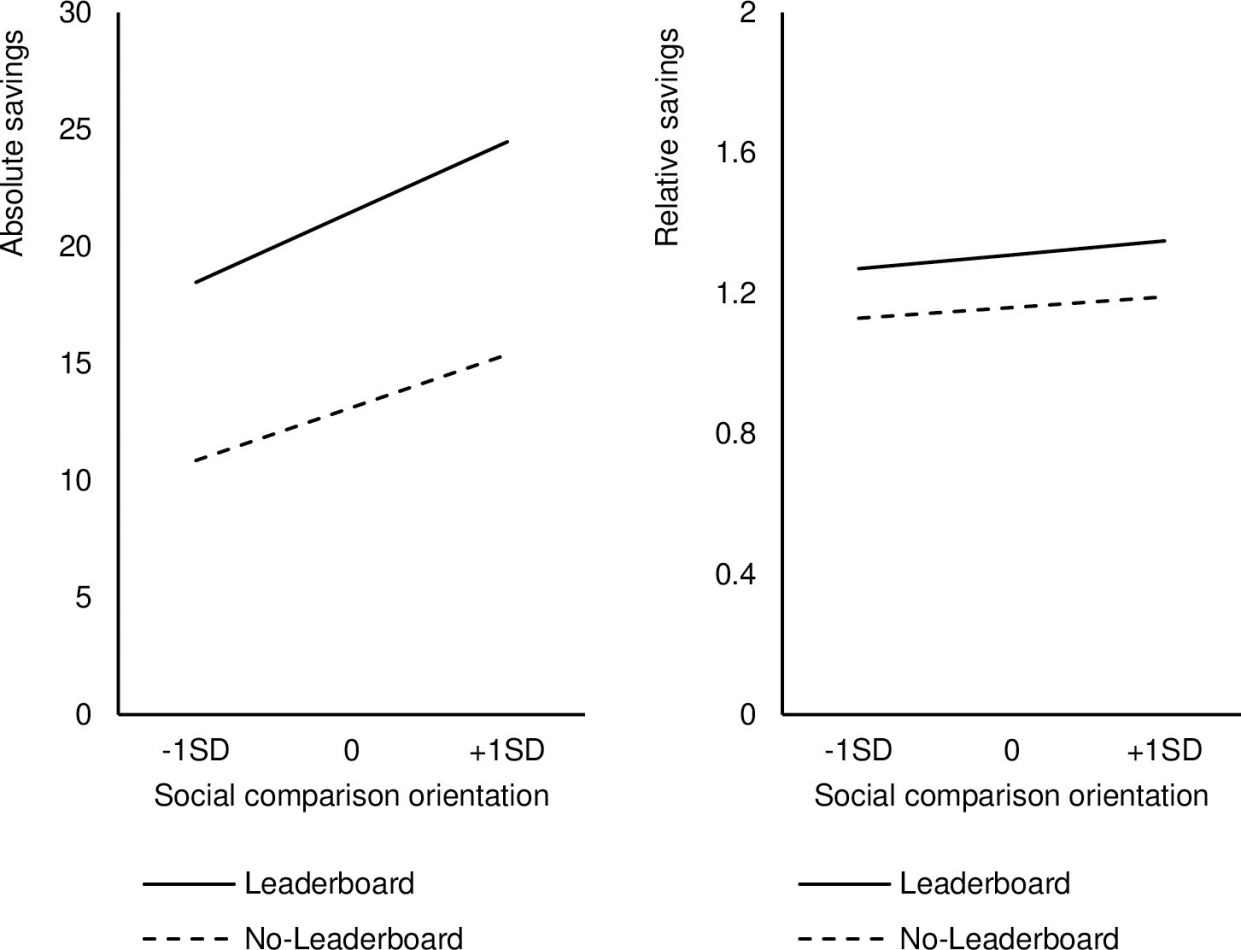
**Absolute savings (left panel) and relative savings (right panel) as a function of the leaderboard and the social comparison orientation.** (Error bars indicate +/–1 Standard Error of the Mean).

On absolute savings, a regression was conducted to test whether the presence of a leaderboard (contrast coded: –0.5 as no leaderboard, 0.5 as leaderboard) increases absolute saving intentions and whether SCO (mean centered) moderates this effect. Replicating the findings of Study 1, the results revealed a significant main effect of the leaderboard, b = 8.35, t = 2.87, p = .004. Participants in the leaderboard condition showed higher absolute savings (M = 21.52, SD = 29.27) than participants in the no-leaderboard condition (M = 13.10, SD = 27.29). The main effect of SCO was not significant, b = 3.51, t = 1.80, p = .07. Still, absolute savings tended to increase with participants’ SCO. The interaction between the leaderboard and the SCO was also not significant, b = 0.99, t = 0.25, p = .80 (Fig 2, left panel).

On relative savings, the results of the regression revealed also for relative savings a significant main effect of the leaderboard, *b* = 0.15, *t* = 3.99, *p* < .001. Participants’ relative savings in the leaderboard condition was higher (*M* = 1.31, *SD* = 0.41) than in the no-leaderboard condition (*M* = 1.16, *SD* = 0.31). The main effect of the SCO was not significant, *b* = 0.05, *t* = 1.86, *p* = .06, but there was a tendency that the more people compared themselves with others, the higher their relative savings were. The interaction between the leaderboard and the SCO was not significant, *b* = 0.02, *t* = 0.36, *p* = .72 (Fig 2, right panel).

Additionally, we repeated the above analyses when including the outliers on the saved amount (Saving 1 and 2). The results differed to some degree but remained in line with our proposition. The main effect of leaderboard became nonsignificant on absolute savings (*b* = 7.96, *t* = 1.32, *p* = .19), but remained significant on relative savings (*b* = 0.07, *t* = 2.25, *p* = .02). On both absolute and relative savings, the interaction between leaderboard and SCO remained nonsignificant (on absolute savings: *b* = 14.61, *t* = 1.82, *p* = .07; on relative savings: *b* = 0.17, *t* = 1.93, *p* = .05; see S2 File for analysis details).

Next, we ran ANCOVAs, accounting for several continuous control variables separately. On absolute and relative savings, the results revealed a positive effect of general attitudes toward saving (*b* = 5.09, *t* = 3.20, *p* = .002; *b* = 0.04, *t* = 1.83, *p* = .07), of importance of saving for retirement (*b* = 4.43, *t* = 2.94, *p* = .004; *b* = 0,04, *t* = 2.05, *p* = .04), and of importance of saving generally (*b* = 4.45, *t* = 2.42, *p* = .02; *b* = 0.05, *t* = 1.97, *p* = .05). Besides, amount of saving each positively was positively correlated with absolute savings (*b* = 1.92, *t* = 2.98, *p* = .003). Age was negatively associated with absolute savings (*b* = –0.26, *t* = –1.93, *p* = .05). Importantly, the pattern of results for both absolute savings and relative savings remained the same after inclusion of the control variables. None of the other control variables (current financial situation and parental financial situation) could explain any of the variance (*p*s > .10).

### Discussion

The results of Study 2 replicate the findings of Study 1 and demonstrate that competitive leaderboards can have a positive effect on people’s intentions to save for retirement. The results show further that individuals’ SCO did not moderate this effect.

## General discussion

People find saving money important and desirable. Despite this, saving money can be challenging. Two studies provide convergent evidence that competitive leaderboards can be an effective tool to increase saving intentions. When a competitive leaderboard was presented (that displayed people’s relative rankings in terms of the amount of money saved for retirement), both relative and absolute saving intentions for retirement increased. The findings further suggest that social comparison standards influence the effect of leaderboards on saving intentions. The results showed that the positive effect of leaderboards was especially pronounced when the standard of upward comparison was high (60%) as compared to low (10%). Study 2 indicated that individuals’ SCO did not moderate the effect of leaderboards on saving intentions. Importantly, the findings persisted after including the control variable, such as attitudes towards saving, employment status, household composition, amount of saving each month, the importance of saving, income, and age.

### Theoretical and practical implications

This research extends research examining the strategies of increasing people’s saving intentions. Most pre-existing strategies to improve saving behavior focus on taking away temptations to spend. For example, commitment devices impose pre-set restrictions to avoid temptations [20]. In addition, automatic transfers of a proportion of someone’s salary into a savings account decreases the amount of money available to spend [9]. Unlike the above strategies to reduce spending, the current research focuses instead on stimulating people to save more. We introduced a simple-to-implement tool to increase saving intentions by making saving fun—gamification. Saving money is usually perceived as unpleasant as it prohibits people from buying desirable products. As we have argued for in the introduction, gamification can make saving money a more pleasant, social, and fun experience. Furthermore, by using leaderboards as a specific type of gamification, the motivation to compete in human nature is elicited through the display of individuals’ rankings based on their savings. As high rankings on leaderboards are generally desirable, competition likely increases people’s motivation to save more.

The present research also extends the effectiveness of gamification into the financial decision-making area. There are several initiatives using gamification to increase savings. For example, users of SavingsQuest app can earn badges when a particular saving goal is reached, leading to a 25 percent increase in savings, as compared to those who do not use this app [57]. In the Long Game app, users can collect more digital coins by saving more money, which can be used to have access to mini-games in which users may win a big amount of money [58]. However, little empirical research has been done to verify the exact factors driving the above effectiveness. The current research focuses on one specific type of gamification, leaderboards, and systematically investigates the effect of social comparison as an important moderating variable specific to this type of gamification. Instead of focusing on saving as an individual endeavor, the current research applies peer influence via leaderboards to improve saving intentions. We believe that this competition, the key game element of leaderboards, plays an important role in increasing saving intentions.

We also believe that this research helps to deepen the understandings of the theory behind gamification. Traditionally, the outcome of gamification has been emphasized, but the underlying processes have been ignored. So far, most research combined several game elements in a gamification design and treated the gamification as a uniform concept. Thus, the most effective game elements to address specific problems and the underlying mechanisms are rather unclear. In this research, therefore, we focused on one specific type of gamification: leaderboards. Leaderboards induce competition among players and provide information (i.e., rankings and corresponding saved amounts) to perform the social comparison, which may drive the effectiveness of leaderboards [32, 59]. Consistent with this idea, the current research indicates that comparison standards indeed impact the effect of leaderboards on saving intentions, suggesting that the social comparison function of leaderboards which is influenced by comparison standards, might impact people’s performance. In addition, though individuals’ SCO does not moderate the effect, their savings intentions increase with individuals SCO in the leaderboard condition, suggesting that the inclination to compete and compare with others may influence the competition and social comparison functions of leaderboards which in turn alters people’s saving intentions.

These findings may have important implications for policymakers and practitioners increasing personal savings. Given the possible severe consequences of saving too little (i.e., financial hardship and poor health), practical and simple-to-implement strategies to increase personal savings are essential. As digital leaderboards can be readily displayed and accessed on a bank app via message boxes or pushed notifications, leaderboards might be a practical tool to increase saving intentions. For example, people can set their savings goals in their bank app. The extent to which people complete their goals may be compared with others in a saving money competition. People can learn about their relative saving performances from the leaderboards. The saving money competition can encourage people to track their savings progress and complete their savings goals as soon as possible.

Leaderboards might be a specifically potent tool for children while developing saving habits. Due to the natural attraction of children to games, gamification is considered to be an effective tool to educate children [60]. Parents can educate children to save money via a saving competition game involving leaderboards. For example, parents can organize a saving money competition among children and their peers. Parents can even be one of the players in the competition. In the competition, children will learn about their relative saving progress from a leaderboard and are motivated to save more. Gradually, children may form a good habit of saving money.

### Limitations and future directions

There are several limitations in our research which suggest avenues for future research. First, we assessed participants’ intentions to save in a hypothetical scenario. Even though intentions generally indicate people’s willingness to enact the behavior and to exert effort in performing it [61], there might still be a gap between intentions and behavior [62]. Future research could examine whether our findings can be extended to actual saving behavior. One possible route would be to organize an actual saving money competition and track whether the implementation of leaderboards encourages people to complete their savings goals and to increase their savings.

Second, the findings of the current research show that the influence of leaderboards on saving intentions was more pronounced when the comparison standard was high (60% more than one’s savings) than when it was low (10% more than one’s savings). Lockwood and Kunda [48] found that unattainable high comparison standards might jeopardize the effectiveness of social comparison. Future research could examine whether leaderboards are ineffective or even counterproductive when the comparison standard is too high (i.e., 80% more than one’s savings). This investigation can provide insights into whether the height of the comparison standard is linearly or curvilinearly related to saving intentions, deepening the understandings of the optimum height of the comparison standard to increase saving intentions via leaderboards.

Third, we did not report findings examining the mediating role that motivation to save might play in the relationship between leaderboards and saving intentions as the analyses suggest that the requirements for meaningful mediation are not all satisfied. Even so, the motivation to improve oneself in competition and social comparison, which refers to increasing savings in our research, is a strong force driving people to perform better in savings [48]. Future research may further examine the role of the motivation to save in the effect using different measures. For example, instead of self-report scales, speed to pursuit a goal may also indicate people’s strength of motivation [63].

Fourth, the current research only examined the effect of leaderboards. Other game elements, such as badges and points, may function as rewards or incentives to motivate saving as well. In line with this idea, badges have been found to be effective in increasing savings [57]. Considering that different game elements function through different mechanisms, one meaningful question to be examined further is which game element works the best to increase savings. This investigation can help identify the most effective gamification strategy in improving savings.

Finally, future research may explore further whether the extent in which people save regularly impacts the effectiveness of leaderboards. The exploratory analysis revealed that the regularity of saving moderated the effect of leaderboards and comparison standards on saving intentions. For people who save regularly, displaying a leaderboard of saved amount increased their saving intentions, and this effect was more pronounced when the standard of upward comparison was high (60%) than low (10%). Whereas for people who do not save regularly, neither the leaderboard nor the standard of upward comparison influenced their saving intentions. Though a sensitive power analysis suggests that our study has enough power to detect the three-way interactions, we should be cautious drawing conclusions given the small sample of irregular savers (*n* = 78). Future research could explore this effect further, looking into the reasons why leaderboards may not be effective for irregular savers. One possible explanation might be that irregular savers do not value savings as much as regular savers do, and therefore do not respond to saving strategies.

To conclude, the current research demonstrated a positive effect of leaderboards on individuals’ financial saving intentions, especially when people compared themselves with a relatively high standard of upward comparison. This may help to address saving difficulties further and improve human financial well-being.

## Supporting information

S1 File Study 1 scenarios. (DOCX)

S2 File Additional analyses of Study 1 and Study 2. (DOCX)

S3 File The influence of the regularity of saving. (DOCX)
