# Supplementary material for: Increasing saving intentions through leaderboards: A gamification approach
Source: PLoS One. 2021 Apr 14;16(4):e0249283. doi: 10.1371/journal.pone.0249283 (PMC8046219; doi:10.1371/journal.pone.0249283)

**S1 File. Study 1 Scenarios**

All participants read this introduction:

You want to start saving money for your retirement. You do not have put money aside for later yet, but you do think it is important. You already opened a savings account, but did not determine how much you will invest every month. From this month on you receive $ 100 extra salary each month. You can use this extra money to save for later. There is no obligation to do so, you can save less or more. It is your choice.

In the no-leaderboard condition with a low/high standard of upward comparison, participants further read:

Now imagine that as of this month your colleagues will also receive $100 extra salary. During the lunch-break, you talk with some colleagues about the importance of saving for your retirement. Your colleagues indicate that they also want to save each month for their retirement. After several weeks you and your colleagues have lunch again and you talk about how much each of you have saved this month. Bob saved most, $X this month (in the low-comparison-standard scenario, X is 1.10 times participants’ Saving 1; in the high-comparison-standard scenario, X is 1.60 times Saving 1).

In the leaderboard condition with a low/high standard of upward comparison, participants further read:

You talk with some colleagues about saving for later during a lunch-break. Just like you, they want to save a monthly amount for their retirement. They also have $ 100,- extra available from this month. Together you decide that you will challenge each other to save more by turning it into a game. Every month a ranking board as shown below will be hung on the wall to see how everyone is doing. This month you are ranked number five on the list. The best saver was Bob and he has saved $X this month (in the low-comparison-standard scenario, X is 1.10 times participants’ Saving 1; in the high-comparison-standard scenario, X is 1.60 times participants’ Saving 1).


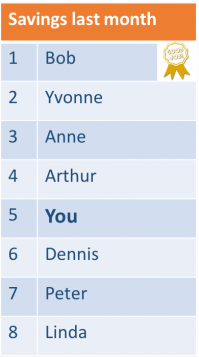

Supplement: S1 File — (DOCX) [file pone.0249283.s001.docx]
