# Supplementary material for: Increasing saving intentions through leaderboards: A gamification approach
Source: PLoS One. 2021 Apr 14;16(4):e0249283. doi: 10.1371/journal.pone.0249283 (PMC8046219; doi:10.1371/journal.pone.0249283)
Supplement: S2 File — (DOCX) [file pone.0249283.s002.docx]

**S2 File. Additional Analyses of Study 1 and Study 2**

**Study 1**

**When including the outliers on the saved amount (Saving 1 and 2), the results remained the same as when excluding participants according to the original criteria** (*N* = 243).

Manipulation check was successful (*p*s < .001). On absolute savings, an ANOVA showed that a significant main effect of the leaderboard, *F*(1, 239) = 4.27, *p* = .04, η_p_^2^ = .02. Absolute savings were higher in the leaderboard condition (*M* = 27.88, *SD* = 82.59) than in the no-leaderboard condition (*M* = 12.11, *SD* = 32.52). There was also a significant main effect of the comparison standard, *F*(1, 239) = 5.75, *p* = .02, η_p_^2^ = .02. Absolute savings were higher in the high-comparison-standard condition (*M* = 30.66, *SD* = 86.64) than in the low-comparison-standard condition (*M* = 10.81, *SD* = 28.61). Besides, the interaction between leaderboard and comparison standard was significant, *F*(1, 239) = 4.54, *p* = .03, η_p_^2^ = .02. Follow-up simple effect tests revealed the same pattern of results as the original analyses (Fig 1, left panel). The effect of leaderboard was not significant in the low-comparison-standard condition (*F*(1, 239) = 0.02, *p* = .96, η_p_^2^ < .001) while was significant in the high-comparison-standard condition (*F*(1, 239) = 8.42, *p* = .004, η_p_^2^ = .03). Besides, in the no-leaderboard condition, there was no significant difference between low-comparison-standard and high-comparison standard, *F*(1, 239) = 0.04, *p* = .85, η_p_^­2^ < .001, while in the leaderboard condition, relative savings of high-comparison-standard condition was significantly higher than that of low-comparison-standard condition, *F*(1, 239) = 10.65, *p* < .001, η_p_^­2^ = .04.

**Fig 1*.* Absolute savings (left panel) and relative savings (right panel) as a function of the leaderboard and the standard of upward comparison (low vs. high)**

On relative savings, consistent with the results for absolute savings, the ANOVA on relative savings revealed a significant main effect of the leaderboard, *F*(1, 239) = 6.74, *p* = .01, η_p_^2^ = .03; relative savings were higher in the leaderboard condition (*M* = 1.34, *SD* = 0.84) than in the no-leaderboard condition (*M* = 1.14, *SD* = 0.28). Also, the main effect of the comparison standard was significant, *F*(1, 239) = 9.04, *p* = .003, η_p_^2^ = .04; relative savings were higher in the high-comparison-standard condition (*M* = 1.37, *SD* = 0.88) than in the low-comparison-standard condition (*M* = 1.13, *SD* = 0.21). The interaction between the leaderboard and the comparison standard was not significant, *F*(1, 239) = 2.30, *p* = .13, η_p_^2^ = .01 (Fig 1, right panel). The effect of leaderboard was not significant in the low-comparison-standard condition (*F*(1, 266) = 0.61, *p* = .44, η_p_^2^ = .003) while was significant in the high-comparison-standard condition (*F*(1, 266) = 8.09, *p* = .01, η_p_^2^ = .03). Besides, in the no-leaderboard condition, there was no significant difference between low-comparison-standard and high-comparison standard, *F*(1, 239) = 1.07, *p* = .30, η_p_^­2^ = .004, while in the leaderboard condition, relative savings of high-comparison-standard condition was significantly higher than that of low-comparison-standard condition, *F*(1, 239) = 10.62, *p* < .001, η_p_^­2^ = .04.

**Study 2**

**When including the outliers on the saved amount (Saving 1 and 2), the results were a little bit different from the results of the original analyses but were still in line with our propositions** (*N* = 384).

Manipulation check was successful (*p* < .001). On absolute savings, a regression analysis showed a nonsignificant main effect of leaderboard, *b* = 7.96, *t* = 1.32, *p* = .19, a nonsignificant main effect of participants’ social comparison orientation (SCO), *b* = –3.44, *t* = –0.86, *p* = .39, and a nonsignificant interaction between leaderboard and SCO, *b* = 14.61, *t* = 1.82, *p* = .07. Further simple effect analysis suggested that at –1SD SCO, the effect of leaderboard was not significant, *b* = –3.03, *t* = –0.36, *p* = .72. However, at +1SD SCO, the effect of leaderboard was significant, *b* = 18.95, *t* = 2.22, *p* = .03; that is, participants in the leaderboard condition had higher absolute savings than participants in the no-leaderboard condition (Fig 2, left panel). This is in line with our proposition that leaderboards are more effective in increasing saving intentions when people are more likely to compare themselves with others.

**Fig 2.** **Absolute savings (left panel) and relative savings (right panel) as a function of the leaderboard and the social comparison orientation**.

On relative savings, the results revealed a significant main effect of leaderboard, *b* = 0.07, *t* = 2.25, *p* = .02. Participants in the leaderboard condition showed higher relative savings (*M* = 1.36, *SD* = 0.58) than participants in the no-leaderboard condition (*M* = 1.21, *SD* = 0.70). The main effect of SCO was nonsignificant, *b* = –0.02, *t* = –0.37, *p* = .71. The interaction between leaderboard and SCO was not significant, *b* = 0.17, *t* = 1.93, *p* = .05. Further simple effect analyses revealed that at –1SD SCO, the effect of leaderboard was not significant, *b* = 0.02, *t* = 0.23, *p* = .82. However, at +1SD SCO, the effect of leaderboard was significant, *b* = 0.28, *t* = 2.96, *p* = .003; that is, participants in the leaderboard condition had higher absolute savings than participants in the no-leaderboard condition (Fig 2, right panel).
