# Supplementary material for: Increasing saving intentions through leaderboards: A gamification approach
Source: PLoS One. 2021 Apr 14;16(4):e0249283. doi: 10.1371/journal.pone.0249283 (PMC8046219; doi:10.1371/journal.pone.0249283)
Supplement: S3 File — (DOCX) [file pone.0249283.s003.docx]

**S3 File. The Influence of the Regularity of Saving**

As the extent in which people save regularly may impact saving intentions, we next analyzed the moderating effect of saving regularity (yes vs. no) for exploratory purposes. A 3-way ANOVA demonstrated that saving regularity moderated the observed interaction between leaderboard and comparison standard on absolute savings, *F*(3, 233) = 4.67, *p* = .003, η_p_^­2^ = .06. For participants who save regularly (*n* = 163), the main effects of the leaderboard *(F*(1, 159) = 25.52, *p* < .001, η_p_^2^ = .14) and of the comparison standard on absolute savings were significant (*F*(1, 159) = 25.61, *p* < .001, η_p_^2^ = .14). In addition, the results revealed a significant interaction between the leaderboard and the comparison standard, *F*(1, 159) = 15.40, *p* < .001, η_p_^2^ = .09 (Fig 1, left panel). Simple effect tests in the low-comparison-standard condition found no difference between the no-leaderboard (*M* = 6.81, *SD* = 17.51) and leaderboard conditions (*M* = 10.46, *SD* = 10.15), *F*(1, 159) = 0.69, *p* = .41, η_p_^2^ = .004. In the high-comparison-standard condition, however, absolute savings were higher in the leaderboard condition (*M* = 39.53, *SD* = 27.82) than in the no-leaderboard condition (*M* = 10.49, *SD* = 24.11), *F*(1, 159) = 37.23, *p* < .001, η_p_^2^ = .19.

For participants who did not save regularly (*n* = 78), however, the main effects of the leaderboard (*F*(1, 74) = 0.08, *p* = .77, η_p_^2^ < .001) and of the comparison standard (*F*(1, 74) = 2.32, *p* = .13, η_p_^2^ = .03), and the interaction between the leaderboard and the comparison standard (*F*(1, 74) = 0.23, *p* = .64, η_p_^­2^ = .003) were all nonsignificant (Fig 1, right panel). Interestingly, this suggests that when people do not save regularly, leaderboards do not have an effect on people’s saving intentions.

**Fig 1. Absolute savings as a function of leaderboard and comparison standard for participants who save regularly (left panel) and for participants who did not save regularly (right panel).**

The same pattern of results was found for relative savings (three-way interaction: *F*(3, 233) = 7.74, *p* < .001, η_p_^2^ = .09). For regular savers, the main effect of the leaderboard (*F*(1, 159) = 32.48, *p* < .001, η_p_^2^ = .17), the main effect of the comparison standard (*F*(1, 159) = 22.55, *p* < .001, η_p_^2^ = .12), and the interaction between the leaderboard and the comparison standard (*F*(1, 159) = 12.87, *p* < .001, η_p_^2^ = .08) were all significant (Fig 2, left panel). In the low-comparison-standard condition, the difference between the leaderboard and no-leaderboard conditions was not significant, *F*(1, 159) = 2.43, *p* = .12, η_p_^2^ = .02; but in the high-comparison-standard condition, the relative savings in the leaderboard condition was significantly higher than that in the no-leaderboard condition, *F*(1, 159) = 39.88, *p* < .001, η_p_^2^ = .20.

However, for participants who did not save regularly, results showed a nonsignificant main effect of the leaderboard (*F*(1, 74) = 0.05, *p* = .83, η_p_^2^ < .001), a nonsignificant main effect of the comparison standard (*F*(1, 74) = 2.93, *p* = .09, η_p_^2^ = .04), and a nonsignificant interaction between the leaderboard and the comparison standard (*F*(1, 74) = 3.17, *p* = .08, η_p_^2^ = .04; Fig 2, right panel).). Simple effect tests showed that the difference between the leaderboard and no-leaderboard condition was nonsignificant regardless of the comparison standard, *p*s > .15.

**Fig 2. Relative savings as a function of leaderboard and comparison standard for participants who save regularly (left panel) and for participants who did not save regularly (right panel).**

Finally, the sensitive power analysis with α = .05, 80% power, *N* = 241, suggested the smallest detectable effect size was η_p_^2^ = .03. Thus, Study 1 had enough power to detect the current significant three-way interaction among saving regularity, leaderboard, and comparison standards on absolute savings and relative savings.
